# Seasonal Metabolic Investigation in Pomegranate (*Punica granatum* L.) Highlights the Role of Amino Acids in Genotype- and Organ-Specific Adaptive Responses to Freezing Stress

**DOI:** 10.3389/fpls.2021.699139

**Published:** 2021-08-12

**Authors:** Parisa Yazdanpanah, Parisa Jonoubi, Mehrshad Zeinalabedini, Homa Rajaei, Mohammad Reza Ghaffari, Mohammad Reza Vazifeshenas, Somayeh Abdirad

**Affiliations:** ^1^Department of Plant Sciences, Faculty of Biological Sciences, Kharazmi University, Tehran, Iran; ^2^Department of Systems and Synthetic Biology, Agricultural Biotechnology Research Institute of Iran (ABRII), Agricultural Research, Education and Extension Organization (AREEO), Karaj, Iran; ^3^Department of Biology, College of Sciences, Shiraz University, Shiraz, Iran; ^4^Improvement Plant and Seed Department, Agricultural and Natural Resources Research and Education Center Research, AREEO, Yazd, Iran

**Keywords:** pomegranate, primary metabolites, frost tolerance, developmental cycle, resistant genotype, fruit tree, stem, bud

## Abstract

Every winter, temperate woody plants have to cope with freezing stress. Winter hardiness is of crucial importance for pomegranate survival and productivity. A comparative morphological and metabolic study was conducted on the stems and buds of 15 field-grown mature pomegranate genotypes in seven time-points during two developmental cycles. Seasonal changes of frost hardiness, as determined by electrolyte leakage method, and metabolite analysis by HPLC and GC revealed the variability in frost hardiness and metabolic contents result from genetic background and organ, as well as seasonal condition. Morphological adaptations, as well as metabolic remodeling, are the distinct features of the hardy genotypes. Larger buds with a greater number of compressed scales and the higher number of protective leaves, together with the higher number and content of changed metabolites, especially amino acids, seem to provide a higher frost resistance for those trees. We recorded two-times the change in metabolites and several-times accumulation of amino acids in the stem compared with buds. A better potential of stem for metabolome adjustment during the hardening period and a higher level of tolerance to stress is therefore suggested. High levels of arginine, proline, glutamine, and asparagine, and particularly the accumulation of alanine, tryptophan, and histidine are responsible for excellent tolerance of the stem of tolerant genotypes. With regard to the protective roles of amino acids, a relation between stress tolerance and the level of amino acids is proposed. This points both to the importance of amino acids in the winter survival of pomegranate trees, and to the evaluation of frost tolerance in other plants, by these specific markers.

## Introduction

Pomegranate (*Punica granatum* L.) is an economically valuable fruit tree that originates from the region spanning Iran to the Himalayas in northern India but is cultivated in many parts of the world today (Holland et al., 2009). Despite a wide geographical distribution and adaptation, its optimal climate conditions are high exposure to sunlight, mild winter with minimal temperature, not lower than −12°C, and dry hot summers without rain, during the last stages of fruit development (Holland et al., 2009). In recent years, pomegranate cultivation and consumption greatly increased due to the dramatic progress of scientific reports on its health-promoting properties (Wu and Tian, 2017; Saeed et al., 2018).

Although Iran is one of the main producers and exporters of pomegranate fruit in the world (Erkan and Dogan, 2018), its production has recently declined due to winter frost damage. In nearly all pomegranate growth and cultivation areas in Iran, minimum winter temperatures may fall to −20°C or even lower. Therefore, winter frost, especially absolute minimum temperature, is considered the major limiting factor in pomegranate yield and productivity. For instance, severe winter frost and unprecedented temperature drops in 2007 and 2016, with the temperature down to −23 and −20°C for 3 and 2 weeks, destroyed 36,931 and 35,000 ha of pomegranate orchards in Iran, respectively. Production declined by 50–90%, in many provinces of Iran (Agricultural Statistics, 2020). According to statistical information from 1990 to 2020, in addition to year-to-year winter frosts, there was a risk of severe winter frost every 10 years in orchards of Iran. Fortunately, Iranian pomegranate germplasm is vastly rich in genetic diversity and more than 760 genotypes from different provinces of Iran have been collected in Yazd Agricultural and Natural Resources Research Center (Zahravi and Vazifeshenas, 2017). A more comprehensive understanding of pomegranate frost hardiness is needed to develop frost-hardy genotypes. The screening and selection of hardier genotypes could be of considerable help in coping with frost problems, enabling producers to reduce severe damage and huge economic losses.

Pomegranate, like other perennial deciduous woody plants, withstands winter temperature by periods of dormancy-activity during the annual cycle. The timing of the hardening/de-hardening process is well-coordinated with the seasonal dormancy-activity cycle and climate changes. Pomegranate growth cessation and entrance to a dormant state start with decreasing photoperiod and temperature. The tree gradually acquires the frost hardiness; reaching its maximum in mid-winter. In response to long days and increasing temperature, frost hardiness declines, and growth resumption occurs in late winter-early spring (Rohde and Bhalerao, 2007). Pomegranate buds develop during the growing season, and their differentiation is completed before the winter dormancy. The vegetative buds of pomegranate are composed of a shoot apex and multiple leaf primordia. A variable number of compactly arranged bud scales protect these primordia from the surrounding environment (Rajaei and Yazdanpanah, 2015).

The adaptive responses of plants to abiotic stress are diverse and involve highly dynamic metabolic and molecular pathways (Krasensky and Jonak, 2012; Preston and Sandve, 2013; Strimbeck et al., 2015; Abdirad et al., 2020). During the hardening process, plants undergo a wide range of structural, physiological, and metabolic modifications which finally lead to the acquisition of a high degree of tolerance against extreme winter temperatures (Preston and Sandve, 2013; Strimbeck et al., 2015). The changes in the level of carbohydrates, amino acids, and the composition of lipids are the most common responses of plants during the process of frost hardiness (Krasensky and Jonak, 2012; Strimbeck et al., 2015).

Frost hardiness shows variability among species, cultivars, genotypes, etc., and varies between different plant organs and tissues, based on developmental stage and plant age, and also along the year (Sakai and Larcher, 1987; Charrier et al., 2013, 2015). The frost hardiness of different plant organs has previously been studied both in gymnosperms and angiosperms: woody stems are more resistant to frost than leaves, vegetative, and reproductive buds (Sakai and Larcher, 1987; Jones et al., 1999; Søgaard et al., 2009; Charrier et al., 2013). On the other hand, the accumulation of metabolites in frost-tolerant plants in different accessions, cultivars, or species differs from those in frost-sensitive examples (Chai et al., 2019). Changes in these metabolites can be related to more tolerance, and are previously documented in other species (Hannah et al., 2006; Krasensky and Jonak, 2012; Chai et al., 2019). Metabolic profiling of more cold-tolerant *Vitis amurensis* and less cold-tolerant *Vitis vinifera* cv. Muscat of Hamburg indicated the involvement of amino acids in the cold tolerance of *V. amurensis* (Chai et al., 2019).

Among the laboratory-based analyses used to assess plant injury, electrolyte leakage (EL) is a common and reliable method of monitoring frost hardiness in different tissues (Charrier et al., 2013; Mayr and Améglio, 2016). This method is based on the principle that damage to cells and loss of membrane integrity results in an enhanced leakage of electrolytes from frost-injured cells. Recording the amount of leakage leads to the estimation of tissue damage (Mayr and Améglio, 2016). EL method is suitable for many woody plants (Jones et al., 1999; Morin et al., 2007; Charrier et al., 2013). The metabolic changes during low temperature stress have been analyzed in some woody plants such as *Camellia sinensis* (Yue et al., 2015) and *Vitis* sp. (Chai et al., 2019). Metabolic time-series experiments have also previously been employed for investigating seasonal changes during the acclimation/de-acclimation cycle in *Arabidopsis* (Kaplan et al., 2004), *Picea obovata* (Angelcheva et al., 2014), and *Camellia sinensis* (Yue et al., 2015).

Despite numerous studies on cold stress responses in woody plants, there is little information on pomegranate responses to frost stress. Ghasemi Soloklui et al. (2012) studied stem frost hardiness among seven 5-year-old pomegranate genotypes during one annual cycle and reported some of the tolerant and sensitive genotypes. They concluded that the content of soluble carbohydrates, especially from fall to mid-winter, is the best indicator of hardiness but, to date, the frost hardiness of mature pomegranate trees has not been reported. In particular, no information is available on the low-temperature tolerance of the different organs of the pomegranate tree, indicating the same or different adaptation to freezing. This comparison between different pomegranate organs extends the study of stress response to the whole tree and provides information on organ-specific frost hardiness (Charrier et al., 2013). This study was conducted to compare frost hardiness of the stem and bud from 15 contrasting pomegranate genotypes in seven time-points during two developmental cycles to select frost-hardier genotypes. We investigated the commonalities and differences of a set of metabolites and their changes between two organs and among contrasting genotypes to identify the necessary compounds and mechanisms that are specific to frost tolerance. We also assessed the correlations between frost hardiness, metabolites, and minimum temperature.

## Materials and Methods

### Sampling Conditions and Plant Materials

Fifteen genotypes from 25-year-old pomegranate trees (*Punica granatum* L.) grown at Yazd Agricultural and Natural Resources Research Center, Yazd province in the central part of Iran (31° 54′ N and 54° 24′ E) were selected from different programs (among 180 and 250 genotypes used in metabolite and morphological programs, respectively) with known frost hardiness characteristics based on expert gardeners' information and preliminary frost hardiness tests.

Full and abbreviation names of genotypes include: “Kadru Shahri Ghasr-e-dasht Fars” (KD), “Rabbab Pust-ghermez Kazerun” (RP), “Pust-siyah Abrandabad Shirin” (PS), “Khafri Jahrom Shirin” (KJ), “Torosh Goli-naz Behshahr” (GN), “Goroch Shahvar Yazdi Shahed” (GO), “Ghahveh-daneh Malas Darjazin” (GD), “Togh-gardan Yazdi Shahed” (TG), “Khusheh-nar Baharestan Sari” (KN), “Bi-tolf Daneh-sefid Malas Ramhormoz” (BT), “Barg-murdi Torosh” (BM), “Bi-hasteh Ladiz Shirin Mirjaveh” (BH), “Shirin Shahvar Yazdi Shahed” (SS), “Vashik Malas Hoshak Saravan” (VH), “Malas1 Hoshak Saravan” (MH).

The fall and winter of 2016–2017 were colder than those of 2015–2016, with mean temperatures of 6.52 and 7.35°C, respectively. The onset of temperatures below zero occurred during some nights in early December 2015 during 2015–2016, and late November 2016 during 2016–2017. The lowest air temperature of −4.1 and −5.3°C occurred in mid-January 2016 and early February 2017, respectively (Supplementary Figure 1). From November to March 2015–2016 and 2016–2017, 8 and 10 days had temperatures below zero, respectively.

Uniform branches (5 mm in diameter) on three different trees of each genotype were sampled from the upper part of the crown during two successive developmental cycles (2015–2017) at the seven different time-points (December 18, 2015, January, 17, October, 1 and December, 15 2016, January 15, February 13, and April 25, 2017). Samples were immediately frozen in liquid N2 for metabolites or packed into plastic bags and kept on crushed ice (4°C) in a cooling box for frost hardiness investigation. Further analysis was performed in the laboratory of Systems and Synthetic Biology, the Department of Agricultural Biotechnology Research Institute of Iran (ABRII).

### Morphological Observations and Visual Assessment

To compare bud samples at successive time points, current branches were collected and their buds viewed with a stereo microscope (Leica MS5, Wetzlar, Germany). Thirty buds from different trees of each genotype were examined. Bud size was measured and the number of their scales was recorded during dissection.

### Frost Hardiness Determination

The frost hardiness of pomegranate stem and bud was determined by the electrolyte leakage test using the method described by Charrier et al. (2013), with slight modifications. The branches were rinsed under running cold deionized water for 15 s and cut into six 5-cm-long segments without bud. Buds were kept intact on 1 mm branch segments. The samples were placed in a moistened tissue and wrapped in aluminum foil per replicate and were transferred to a temperature-controlled freezer (Electrosteel, Tehran, Iran) with a cooling rate of 2°C/h down to −10, −15, −20, and −25°C and either −5°C in spring and −30°C in winter. Each target temperature was maintained for 2 h and then samples were withdrawn and thawed overnight at 5°C. A control sample was stored at 5°C and another control was kept in a freezer at −80°C.

After freezing treatment, branches were cut into 5-mm-long segments and five buds per temperature were used. Samples were transferred into conical tubes with 15 mL of distilled-deionized water. Tubes were shaken for 24 h before the first electrical conductivity (EC1) measurement. Samples were autoclaved at 120°C for 45 min to allow maximum leakage and cooled down to room temperature and EC2 was measured again. Relative electrolyte leakage (REL) was calculated using the formula: REL = (EC_frozen_ − EC_water_) × 100/(EC_autoclave_ − EC_water_).

### Metabolite Measurements

Soluble carbohydrates were extracted and measured according to Abdirad et al. (2020). Freeze-dried samples (100 mg) were finely ground using a mixer mill (Retsch, MM200). Samples were extracted three times with 80% ethanol followed by centrifugation at 5,000 rpm for 10 min and the aqueous phase was dried at 40°C. Then, 10 mL distilled water, 0.47 mL barium hydroxide (0.3 N), and 0.5 mL zinc sulfate (ZnSO_4_) (5%) were added followed by centrifugation at 5,000 rpm for 10 min. The supernatant was evaporated and the pellet was re-dissolved in HPLC grade distilled water and filtered through 0.45 m filters, before application to a reversed phase HPLC (Knauer, Germany) on a Eurokat H column (300 × 80 mm, 10 μm particle size). Acidic water (containing sulfuric acid, pH = 2.5) with a flow rate of 1 mL/min was used as the mobile phase. Sucrose, Glucose, and fructose were detected by the RI detector, and the peak areas were used to determine sugar contents based on their corresponding standards. Soluble carbohydrate concentrations were finally calculated through a calibration curve and expressed as milligrams/gram dry weight.

Amino acids were extracted by adding 1 mL of 80% ethanol to 100 mg of freeze-dried samples followed by heating at 80°C for 60 min and then centrifugation at 13,000 rpm for 10 min. A solution containing 50 mg o-phthaldialdehyde (OPA), 4.5 mL MeOH and 0.5 mL borate buffer (50 mM, pH = 9.5), and 50 μL of 2-mercaptoethanol was prepared. One hundred microliter of OPA solution and 200 μL borate buffer were added to 250 μL of the concentrated supernatant and kept for 2 min at room temperature. The reaction was quenched by adding 50 μL hydrochloric acid (HCl) to the solution. Amino acids were separated on a C18 column (HALO C18, 4.6 × 50 mm, 5 μm particle size, Advanced Materials Technology, Wilmington, USA), by HPLC. Amino acids were detected by RF-10 AXL fluorescence detector (Berlin, Germany) with excitation and emission at 330 and 450 nm, respectively. A gradient of 20–70% MeOH in sodium acetate (NaOAc) buffer (50 mM, pH = 7.0) with a flow rate of 1.1 mL/min was used as mobile phases. The amount of each amino acid was calculated by comparing its peak area with that of the corresponding standards (Abdirad et al., 2020). Amino acid concentrations were finally calculated through a calibration curve and expressed as milligrams/gram dry weight.

Fatty acids were extracted and measured according to Talebi et al. (2013). Briefly, 500 μl of the extraction buffer containing methanol (MeOH) and 2% of concentrated H2SO4 was added to 100 mg of freeze-dried samples and the samples were incubated (2 h, 80°C, 750 rpm). Three hundred microliter of 0.9% NaCl solution and 150 μl of hexane were added into the reaction tube, respectively. Then, the samples were centrifuged (3,000 rpm, 25°C, 5 min). Finally, the supernatant containing the hexane and fatty acid methyl ester (FAME) was used for GC analysis. The fatty acid determination was carried out on a Varian CP-3800 GC (Varian, Inc., Palo Alto, CA) equipped with a CP-Sill 88 fused silica column (100 m, 0.25 mm I.D., film thickness 0.25 μm). The oven temperature was maintained at 130°C for 4 min, then programmed to increase to 180°C at a rate of 5°C/min, and kept at this temperature for 8 min. Finally, the oven temperature was programmed to rise from 180 to 220°C at a rate of 4°C/min under the following conditions: carrier gas Helium (1 mL/min), split ratio 20:1, and flame ionization detector (FID) 280°C. Fatty acid peaks were identified by comparison of the retention time with FAME standards. Standards of fatty acids (including C16:0, C16:1, C18:0, C18:1, C18:2, C18:3), were purchased from Merck Company (Darmstadt, Germany).

### Statistical Analysis

Frost hardiness was expressed as LT50 by fitting response curves with the following logistic sigmoid function (Ghasemi Soloklui et al., 2012):

$$\begin{array}{l} R=\frac{a}{1+e^{b\left(x-c\right)}}+d \end{array}$$

where R was REL; x was treatment temperature; b was the slope of the function at the inflection point c; and a and d determine the upper and lower asymptotes of the function, respectively.

The differences between values of LT50 and metabolite contents of stem and bud samples at each time-point were assessed with a three-way analysis of variance (ANOVA) and means were separated using LSD (*p* ≤ 0.05). Comparative heat maps, principal component analysis (PCA), partial least squares-discriminant analysis (PLS-DA), and correlation heatmaps were performed using the Metaboanalyst (5.0) online analysis software (www.metaboanalyst.ca/). The data were log_2_ transformed and normalized before analysis.

## Results

### Frost Hardiness Displayed Diversity Among Genotypes and Between Two Organs

**Figure 1:**
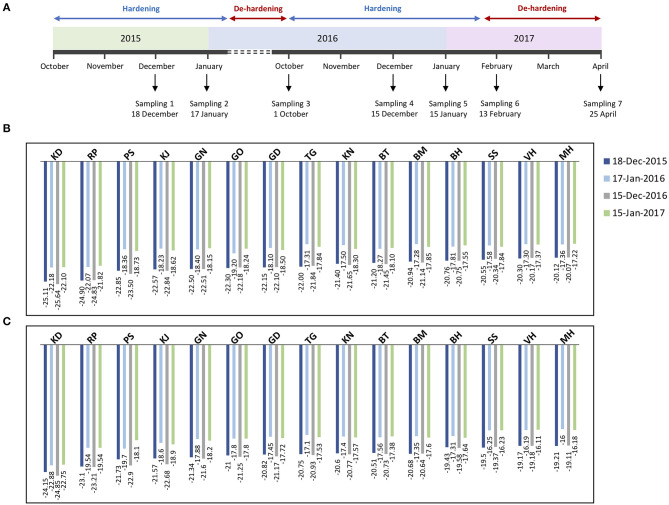
Timeline of sample collection of pomegranate genotypes during two successive developmental cycles **(A)**. Stem and bud samples were collected at seven time-points (December 18, 2015, January, 17, October, 1 and December 15, 2016, January 15, February 13, and April 25, 2017), in coordination with the timing of the tree hardening/de-hardening cycle. Frost hardiness (expressed as LT50) of the stem **(B)** and bud **(C)** of fifteen pomegranate genotypes (KD, RP, PS, KJ, TG, GO, GD, BT, KN, GN, BM, BH, SS, VH, and MH) during December and January of 2 successive years.

For differential sampling and screening, we chose seven time-points during two successive developmental cycles, that are coordinated with the timing of the hardening/de-hardening cycle (Figure 1A). EL method allowed us the calculation of lethal temperature at which 50% of the total ion leakage occurs (LT50) and the evaluation of frost hardiness dynamics in the pomegranate tree. Although the changes of frost hardiness were recorded throughout the pomegranate annual cycle, we selected LT50 values of winter months, i.e., December and January of 2 successive years for representing the maximum frost tolerance of the two organs of each genotype (Figures 1B,C).

Overall, changes of frost hardiness were similar among the fifteen genotypes and between two organs. The frost hardiness level increased gradually during fall, reached a maximum level in December and January, and decreased again toward April. LT50 values ranged from −25.11 to −20.12°C and −25.64 to −20.07°C for stems and ranged from −24.15 to −19.21°C and −24.85 to −19.11°C for buds in December 2015 and 2016, respectively. The hardening/de-hardening cycle was well-accorded, with air temperature values in both years; a lower LT50 value was recorded for almost all the genotypes during winter 2016–2017, compared with winter 2015–2016, due to colder air temperatures and more freezing days (Supplementary Figure 1). One and two weeks before the second sampling (i.e., December), 4 and 7 days and nights with temperatures below zero occurred during 2015–2016 and 2016–2017, respectively.

Between the two investigated organs, the stems are hardier than the buds in both years (Figures 1B,C). Although frost hardiness of the bud was higher than the stem in October for all the genotypes, the stem showed higher frost hardiness than the bud during winter and also was de-hardened later during February and April (*p* ≤ 0.05 for almost all the genotypes).

Among the genotypes, KD (−25.11 and −25.64°C for the stem, −24.15 and −24.85°C for bud) and RP (−24.90 and −24.83°C for the stem, −23.10 and −23.21°C for bud) had significantly lower LT50 values and therefore the highest frost hardiness during December 2015 and 2016, respectively. KD and RP maintained high frost hardiness during winter and had a low de-hardening rate toward April. On the other hand, the lowest frost hardiness was recorded for MH (−20.12 and −20.07°C for the stem, −19.21 and −19.11°C for bud) and VH (−20.30 and −20.17°C for the stem, −19.17 and −19.18°C for bud) during December 2015 and 2016, respectively. Other genotypes were considered as the intermediate in frost hardiness level (Figures 1B,C). We designed three distinct groups based on LT50 data in the present experiment and also based on expert gardeners' information and preliminary frost hardiness test, as well as according to Ghasemi Soloklui et al. (2012): the frost-hardy group included KD and RP, the frost-sensitive group contained VH and MH, and the intermediate group covered other genotypes: PS, KJ, TG, GO, GD, BT, KN, GN, BM, BH, and SS.

### Morphological Adaptations Support Pomegranate Tree Against Frost Stress

KD and RP, as the hardiest genotypes, had normal growth during the subsequent spring and early summer. MH and VH were the least hardy genotypes and suffered more winter injury with frost signs including reduced growth, bud necrosis, formation of small leaves, and leafless terminal branches with the frozen state. Hardy genotypes had larger buds while, in resistant genotypes, buds were smaller. Our microscopic observations under a stereo microscope revealed variability in the number of scales among genotypes. The buds of hardy genotypes had more scales (6–8) that were tightly packed with primordia but in the sensitive genotypes there were fewer scale numbers (2–4). The scales were not completely packed together. The buds of hardy genotypes had more sclerenchymatous leaves (scaly leaves) than those of sensitive genotypes during dissection by removing scales.

### Seasonal Metabolite Analysis Revealed Differential Responses of Two Organs and Different Genotypes

The stem and bud of 15 pomegranate genotypes at seven time-points during two developmental cycles were used for three soluble carbohydrates, twenty-two amino acids, and six fatty acids extraction followed by HPLC and GC analysis. Despite the similarity of the rhythm of seasonal changes, the content of investigated metabolites varied depending on the genotype, organ, and sampling date.

**Figure 2:**
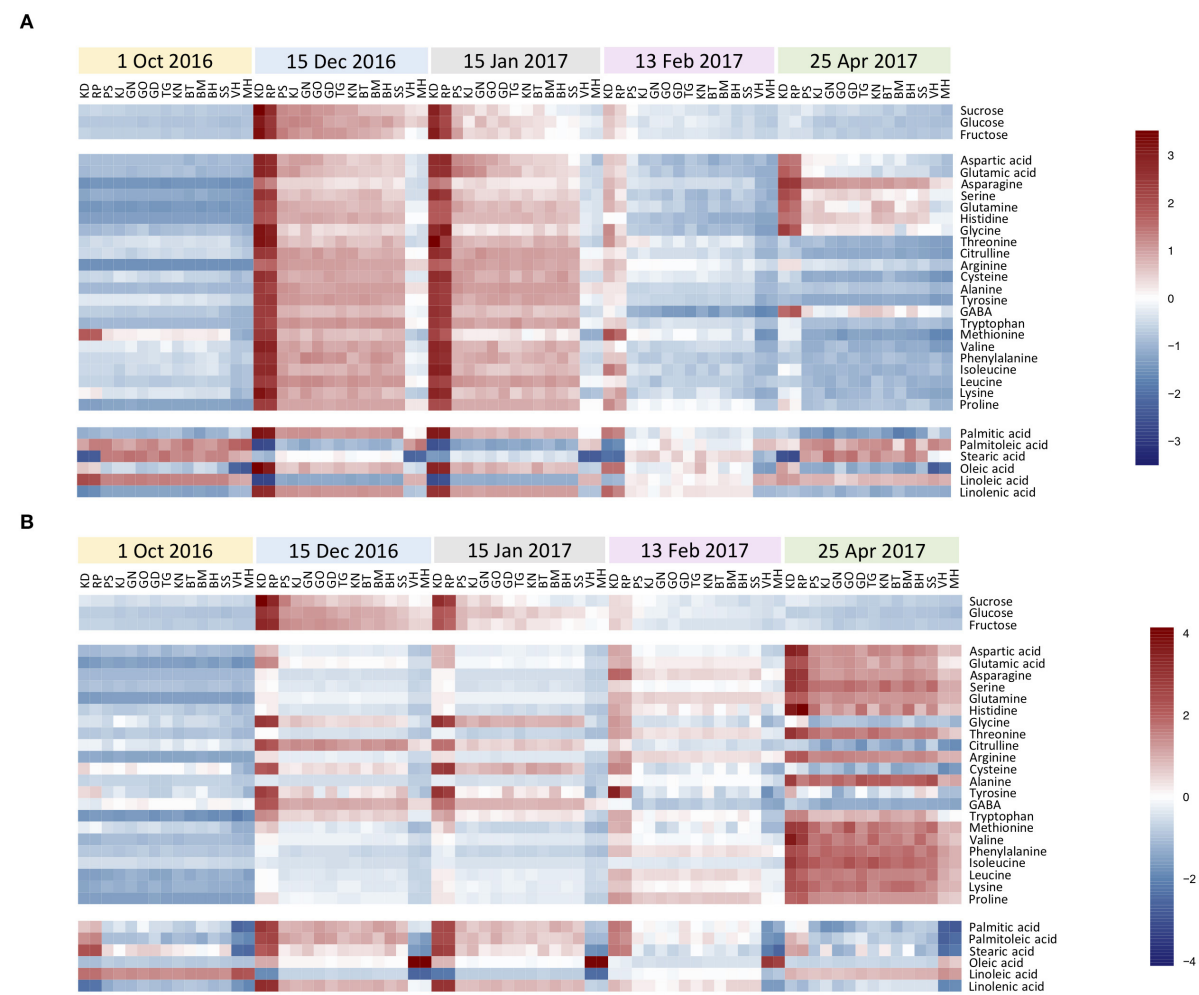
The heatmaps for seasonal variation of primary metabolites in pomegranate stem **(A)** and bud **(B)**. The column represents each genotype (KD, RP, PS, KJ, TG, GO, GD, BT, KN, GN, BM, BH, SS, VH, and MH) at the specific time-point (1 October and 15 December 2016, 15 January, 13 February, and 25 April 2017). Each cell represents the mean values of three biological replicates of each metabolite. The color scales from blue (low) to red (high) and is proportional to the normalized values (expressed as log_2_) of the concentration of each metabolite.

Figures 2A,B shows a heatmap with the normalized values (expressed as log 2) of investigated metabolites for each genotype during the annual cycle in pomegranate stem and bud, respectively. Four distinct patterns of seasonal changes were recorded for investigated metabolites during the annual cycle: (a) an increase toward December and January, a decline in February, and another rise toward April. This cluster contained eight amino acids in the stem. Metabolites in cluster (b) increased rapidly toward December and January and then declined gradually toward April. This cluster included three soluble carbohydrates and some fatty acids in both stem and buds, and fourteen amino acids in the stem. Metabolites in cluster (c) tended to increase toward December and January and kept their rise toward February and April. This cluster contained the majority of amino acids in the buds. Metabolites in cluster (d) showed a distinct decrease toward December and January and then increased in February and April, including only some fatty acids in both stem and buds. Stearic acid was the only metabolite that represented diffuse seasonal pattern among genotypes and between organs: stem of hardy genotypes and buds of sensitive genotypes were placed in cluster (b) while, the stem of intermediate and sensitive genotypes and buds of hardy and intermediate genotypes were placed in cluster (d) (Supplementary Tables 1, 2).

In general, a higher number of metabolites were changed in the stem compared with the buds during the hardening period (20 vs. 10 metabolites). Metabolites that exhibited the most noticeable increase in the stem of all the genotypes during the hardening period were arginine (19 to 29-fold increase), alanine (9.3–20.7 ×), proline (11–20.3 ×), tryptophan (7–19.7 ×), glutamine (5.2–17 ×), asparagine (5–13.7 ×), histidine (6.3–12.5 ×), glucose (4–8.4 ×), fructose (2.6–8.5 ×), glutamic acid (2.2–8 ×), sucrose (3.7–7.9 ×), aspartic acids (2–7.8 ×), serine (3–7.5 ×), glycine (2–6.5 ×), leucine (2.5–5.5 ×), linolenic acid, phenylalanine and isoleucine (2–5 ×) while, palmitoleic acid (2–6 ×) and linoleic acid (2–4 ×) showed the decline during hardening period. In the buds, glutamine (6–12 ×), arginine (6–10.7 ×), proline (5–10.6 ×), fructose (4.3–8.9 ×), glucose (4.8–7.8 ×), asparagine (3–7 ×), sucrose (2–6 ×), glutamic acid (2.5–4.7 ×), oleic acid (2.2–4 ×), and aspartic acids (1.6–4 ×) were the metabolites which exhibited the most rise during hardening period. Linoleic acid (2.2–3.5 ×) was the only metabolite that represented a decrease in buds during this period.

The fold change of soluble carbohydrates was similar in hardy and sensitive groups in both organs during the hardening period. The stem showed more increase in sucrose, as compared to the buds. The fold change of glucose was almost similar in both stem and bud. The buds exhibited more rise in fructose, as compared to the stem. Amino acid groups exhibited considerable accumulation in both tolerant and sensitive genotypes; however, their increase in the stem was two to several times that of the rise in the buds in both groups of genotypes. The extent of amino acid accumulation in the hardy genotypes was several times that of the increase in the sensitive genotypes. The fatty acid changes (increase or decline) occurred more in the stem of tolerant genotypes than in the buds. This was reversed in the sensitive genotypes.

Comparing all the genotypes, the frost-hardy genotype (KD) showed the greatest fold change of soluble carbohydrates, amino and fatty acids, while frost-sensitive genotypes (MH and/or VH) had the lowest fold change in both organs during 2 years (Supplementary Tables 1, 2).

### Metabolite Profile Was Impacted by Genotype and Organ

**Figure 3:**
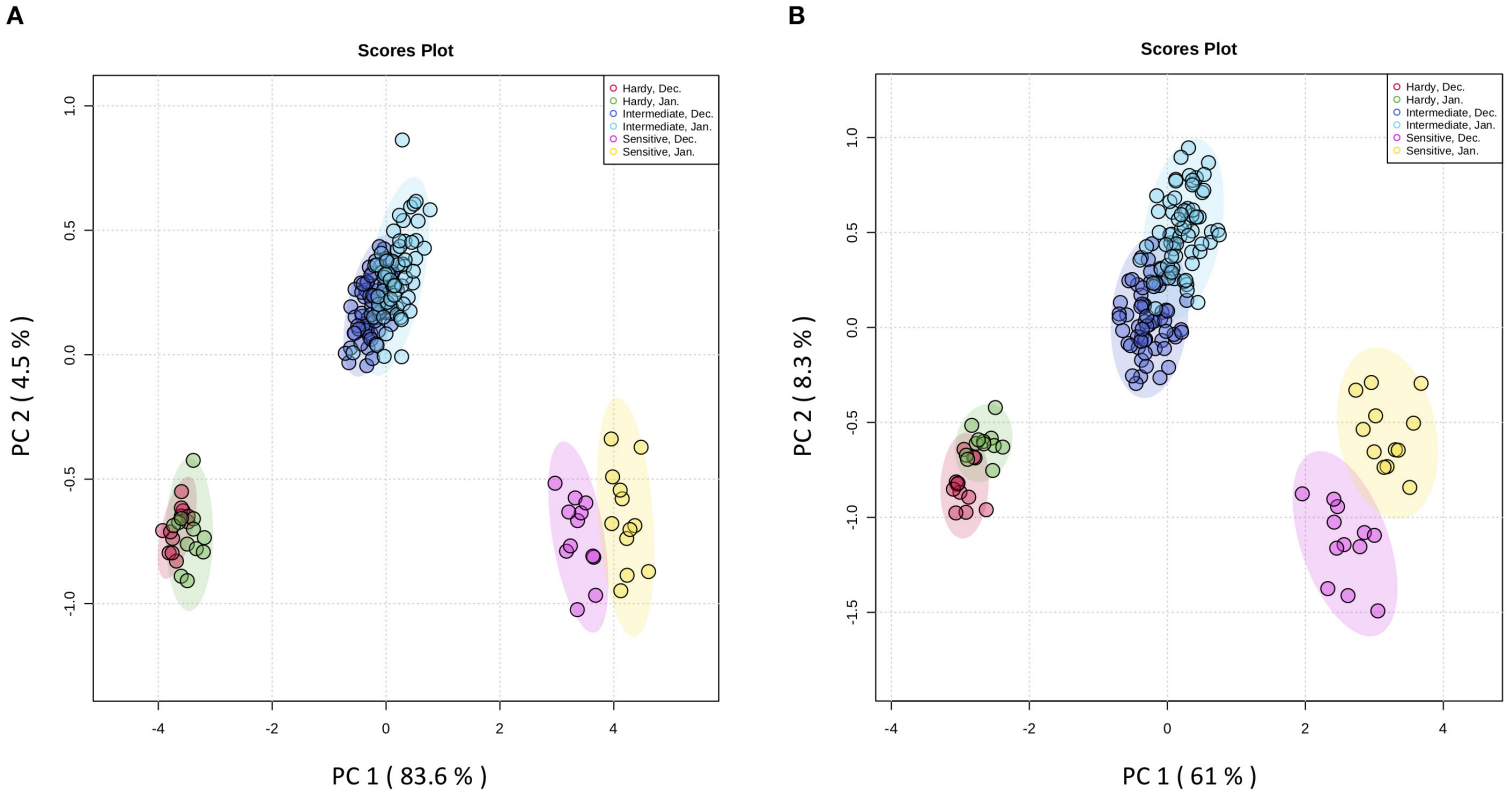
Score plots from PCA of metabolites in stem **(A)** and bud **(B)** of fifteen pomegranate genotypes in December and January of two developmental cycles. The first two principal components (PC1 and PC2) are shown. Stem and bud samples in each time-point are represented by circles with different colors. Each point represents an individual biological replicate (*n* = 3). Samples were segregated with regards to genotype and time-point. Red circles: samples from hardy genotypes in December; green circles: samples from hardy genotypes in January; purple circles: samples from intermediate genotypes in December; blue circles: samples from intermediate genotypes in January; pink circles: samples from sensitive genotypes in December; yellow circles: samples from sensitive genotypes in January.

The principal aims of the present study were to identify frost-hardier genotypes and the key metabolites related to frost tolerance. We selected two time-points i.e., December and January for multivariate data analysis when pomegranate trees were fully hardened. To reveal the effect of the hardening process on metabolite modification, the metabolite data sets derived from the stem and bud samples of the 15 genotypes during December and January of 2 successive years were independently subjected to principal component analysis (PCA) (Figure 3 and Supplementary Figure 2 and Supplementary Table 3). The PCA score plot for both organs showed that principal component 1 (pc1; 83.6 and 61% of the variance for stem and bud, respectively) separated data with regard to genotype and time-points of sampling. The PCA results confirmed our designation based on LT50 data. In both organs, PC1 separated three groups of genotypes: the frost-hardy group including KD and RP; the frost-sensitive group containing MH and VH; and the intermediate group, which covered other genotypes.

**Figure 4:**
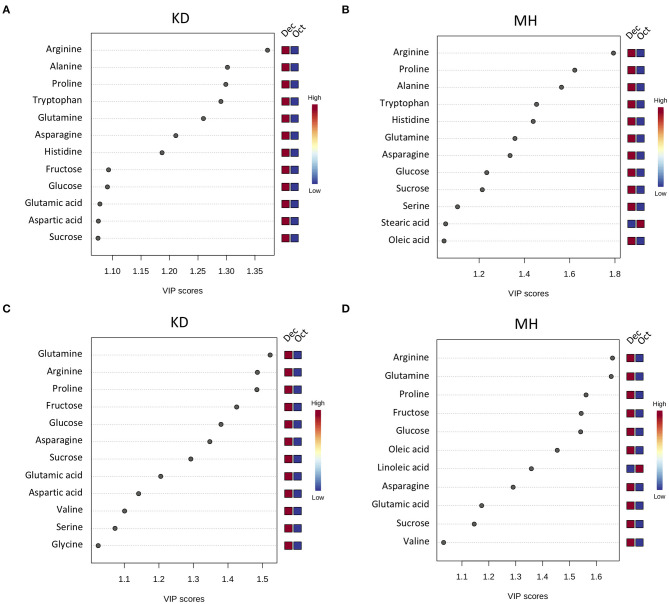
Variable importance in projection (VIP) plots with the important metabolites in response to frost stress identified by PLS-DA analyses in descending order of importance in the stem **(A,B)** and the bud **(C,D)** of KD and MH. The colored boxes on the right indicate the relative concentrations given on a scale of high (red) to low (blue) for the corresponding metabolite in each group.

To identify the frost-responsive metabolites in the stem and buds of pomegranate, the partial least squares-discriminant analysis (PLS-DA) was performed on KD (hardiest genotype) and MH (the most sensitive genotype) for data from October (control or pre-hardening stage) and December (hardening stage), separately. Frost stress explained 98.4 and 85.4% of the total variation in stem (Supplementary Figure 3 and Supplementary Table 4) and 90.5 and 74.4% of the total variation in the bud (Supplementary Figure 4 and Supplementary Table 4) of KD and MH, respectively. Based on a VIP (variable importance in projection) score of >1, the important metabolites involved in frost stress were identified in the stem and bud of KD and MH genotypes. Amino acids seem to contribute to the stem hardening, while in the bud, soluble carbohydrates (fructose and glucose) and fatty acids (oleic and linoleic acid) were found as important metabolites together with amino acids (Figure 4).

We also performed correlation analyses on LT50 and the metabolite data sets derived from the stem and bud samples of the 15 genotypes (Supplementary Tables 5, 6). The correlations between frost hardiness, investigated metabolites and minimum temperature (T_min_) are shown in Supplementary Figure 5.

### Hardening Process Is Associated With Remodeling of the Primary Metabolism

**Figure 5:**
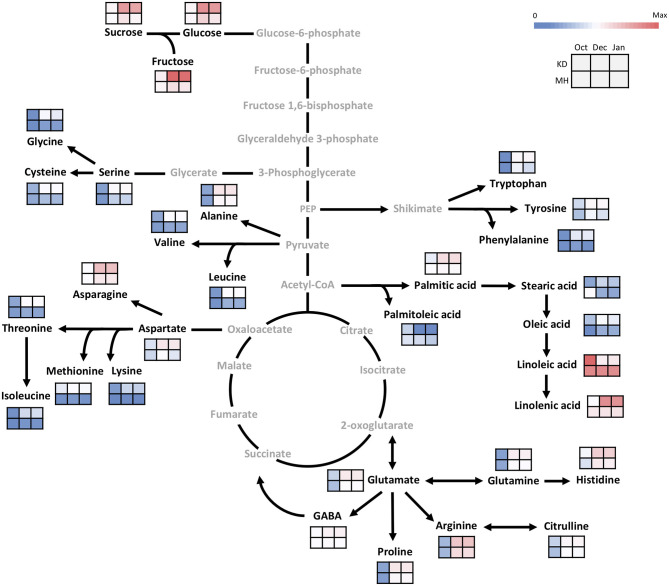
Changes in the primary metabolism in the stem of KD and MH during the hardening process from October to January. Quantified metabolites are set in black and non-measured metabolites are set in gray color. Each heat map represents the intensity of the corresponding metabolite at three time-points (October, December, and January) of hardy (KD) and sensitive (MH) genotypes; October (control or pre-hardening stage), December, and January (hardening stage).

In October, as the pre-hardening stage, the difference of metabolite contents between the stem of KD and MH was below 1.5 times except for methionine, oleic acid, stearic acid, lysine, sucrose, and linolenic acid. The difference in metabolite values between the two cited genotypes became evident toward December and January 2016–2017 (Figure 5). The content of amino acid lysine in the stem of KD was 5.7 times that of MH while, palmitoleic acid in MH was 6.5 times that of KD. Fructose, methionine, aspartic and glutamic acid amounts in KD were 4–5 times that of MH. Linoleic acid amount in MH was 3.5 times that of KD. Sucrose, serine, glycine, threonine, cysteine, valine, phenylalanine, and isoleucine values in KD were 3–4 times that of MH. Glucose, asparagine, glutamine, histidine, citrulline, alanine, GABA, tryptophan, tyrosine, leucine, proline, oleic, and linolenic acid contents in KD were 2–3 times that of MH. Arginine, palmitic, and stearic acids showed a 1.5 time increase in content in KD, as compared to MH.

**Figure 6:**
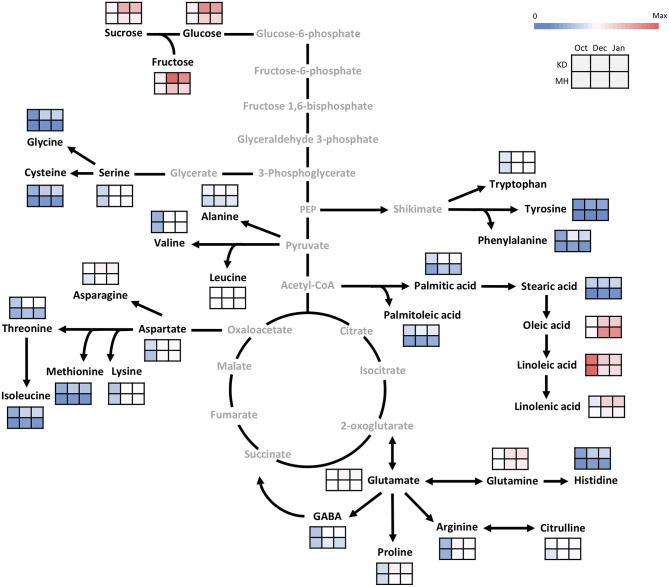
Changes in the primary metabolism in the bud of KD and MH during the hardening process from October to January. Quantified metabolites are set in black and non-measured metabolites are set in gray color. Each heat map represents the intensity of the corresponding metabolite at three time-points (October, December, and January) of hardy (KD) and sensitive (MH) genotypes; October (control or pre-hardening stage), December, and January (hardening stage).

In the buds, similar to the stem, the difference in metabolite values between KD and MH was below 1.5 times in October except for stearic acid, cysteine, methionine, palmitoleic acid, palmitic acid, isoleucine, tyrosine, histidine, glycine, sucrose, and glucose. An increase in the content of metabolites was observed in pomegranate buds toward December and January, but their rise was very low, compared to the stem (Figure 6). The sucrose, glycine, aspartic acid, and phenylalanine amounts in KD were 3–4 times that of MH. Glucose, fructose, glutamic acid, asparagine, histidine, threonine, arginine, cysteine, tyrosine, methionine, isoleucine, proline, palmitoleic, and stearic acids content in KD were 2–3 times that of MH.

## Discussion

### Pomegranate Tree Responses to Frost Stress in a Time-Dependent Manner

Pomegranate trees established the dormancy and hardened gradually during fall, and reached maximum hardiness in mid-winter and de-hardened toward late winter or early spring, in coordination with bud-break and new growth resumption. Concerning the same sampling dates in 2 successive years, we concluded that observed differences in hardiness come from climate changes, as reported by Szalay et al. (2017) in *Prunus domestica* cultivars.

In the stem and bud of all the pomegranate genotypes, the sucrose, glucose, and fructose contents increased during fall, reached their peak in mid-winter, responsible for a high degree of freezing tolerance, and then declined gradually toward spring. It seems that the accumulation of the hexose including glucose and fructose, and disaccharide sucrose is a common response during cold acclimation (Kaplan et al., 2004; Bocian et al., 2015; Yue et al., 2015; Chai et al., 2019). Sugars function as osmo-protectants and stabilize the biological membranes. They are essential for the repair of freeze-induced cavitated vessels and facilitate deep supercooling at high levels (Krasensky and Jonak, 2012; Strimbeck et al., 2015; Mayr and Améglio, 2016). In the vegetative buds of *Picea abies*, primordial cells close to the ice barrier tissue accumulated an amorphous starch and di, tri, and tetra-saccharides (Kuprian et al., 2017).

All the amino acids in the stem and buds of all pomegranate genotypes increased toward December and January but three different patterns were observed toward spring: (a) metabolites which declined in February and raised again toward April, and all recorded in the stem. (b) metabolites which declined in February and April and were related to both stem and bud. (c) metabolites which kept their rise in content during February and April and were all registered in buds. Bocian et al. (2015) and Andersen et al. (2017) stated that the accumulated amino acids are not only involved in the plant survival during unfavorable conditions but can also be an important source of nitrogen and carbon required for plant resuming growth. Increasing levels of proline in developing buds can be related to its important role in flower development (Andersen et al., 2017).

Although the majority of amino acids accumulated in the stem and buds of all the pomegranate genotypes during the hardening period, most accumulations were registered for arginine, proline, glutamine, and asparagine in both stem and buds, and alanine, tryptophan, and histidine in the stem, but not in the buds. The similarity of metabolite changes between pomegranate and other plant species (Kaplan et al., 2004; Angelcheva et al., 2014; Bocian et al., 2015; Chai et al., 2019) can be related to conserved mechanisms of metabolic adjustment to increase cold tolerance (Chai et al., 2019).

Here, an increase of proline and arginine was found in the stem and buds of all the genotypes. A large body of data has described proline accumulation under stress conditions and its function as a compatible osmolyte, stabilizer of proteins, membranes, and subcellular structures, regulator of redox potential, molecular chaperone, radical scavenger, and signaling molecule (Szabados and Savouré, 2010; Krasensky and Jonak, 2012; Casartelli et al., 2018). Arginine is a suitable storage form for organic nitrogen because of its high nitrogen to carbon ratio, which is a precursor for the biosynthesis of proline, and also a precursor for nitric oxide (NO) and polyamines, which play crucial roles in regulating abiotic stress (Winter et al., 2015).

Our results also demonstrate that an accumulation of large amounts of glutamine, asparagine, glutamic, and aspartic acids during the hardening process. These metabolites may be used by the plant as an ammonium donor for the synthesis of other amino acids (Glutamic acid and glutamine), an active ammonium donor, and a precursor for the biosynthesis of pyrimidine and NAD (aspartic acid) and transport/storage compounds (asparagine) (Hincha et al., 2012; Casartelli et al., 2018).

Large amounts of alanine, tryptophan, and histidine were preferentially accumulated in the pomegranate stem. Other reports have proved that alanine acts as an osmo-protectant and can restore the activity of lactate dehydrogenase damaged by frost/defrost rounds (Bocian et al., 2015). The accumulation of tryptophan can be related to important roles in osmotic adjustment, stomatal regulation, and ROS scavenging under stress conditions (You et al., 2019).

The results presented in this study demonstrated a significant increase in the content of linolenic and oleic acids, but a decrease in linoleic acid, in both organs of all the pomegranate genotypes during the hardening stage. The changes of palmitoleic acid were different based on organ. The level of stearic acid differed not only between both organs but also changed among different groups of genotypes. The concentrations of oleic, linoleic, and linolenic acids changed notably from pre- to full-acclimation stages in *Picea obovata* needles (Angelcheva et al., 2014). Zhang et al. (2020) concluded that the accumulation of large amounts of linolenic acid in peanut plants under cold stress can be related to fatty acid b-oxidation and thus jasmonate biosynthesis which through CBF-dependent signaling can improve plant cold tolerance. Changes in fatty acids composition and accumulation of long-chain mono- and polyunsaturated fatty acids help to maintain membrane fluidity, prevent membrane phase change, and are important for regulating the activity of membrane-associated enzymes during frost hardening (Angelcheva et al., 2014; Strimbeck et al., 2015; Zhang et al., 2020).

### Differential Responses of the Stem and Bud to Frost Stress Reveal Resistance Variability Within Pomegranate Tree

Higher frost hardiness in winter and a slow rate of de-hardening at spring in the stem, allowed us to conclude that the stem is frost hardier than the buds, similar to the findings in some gymnosperms and angiosperms (Jones et al., 1999; Søgaard et al., 2009; Charrier et al., 2013). The differences in organ-specific tolerance result from the different mechanisms plants use in response to low temperatures. In grape, buds avoid extra-cellular freezing and supercool, whereas canes experience extra-cellular freezing (Jones et al., 1999).

In walnut trees and *Hedera helix* L., bark, and cambium were relatively susceptible to frost during the growing season but during winter, they were able to harden more than xylem parenchyma (Andergassen and Bauer, 2002; Charrier et al., 2013). Conversely, buds hardened but remained more sensitive than other parts during winter. The difference in cold tolerance may be explained by the anatomical characteristics of stem bark and cambium, as compared to bud. On the other hand, frost has a remarkable impact on wood or meristematic tissues but has a low effect on bark (Charrier et al., 2013). Leaf primordia are the least differentiated and most sensitive parts of bud throughout the year (Andergassen and Bauer, 2002). Therefore, buds are more exposed to freezing events however, the bud scales and cuticle may play an essential role as water or ice proof structures (Kuprian et al., 2017).

Although buds are typically more sensitive than the stems in pomegranate, there is a difference in bud resistance between the genotypes: hardier genotypes have larger buds with more protective scales while, in less resistant genotypes, buds are smaller and the number of protective scales is lower. Therefore, we assumed the assortment for buds of pomegranate genotypes. A decrease in water content of the shoot primordia and reduction in their size in response to decreasing temperature (Ide et al., 1998) may explain the smaller size of buds in less frost resistant genotypes.

The seasonal progress of frost hardiness differs between the two organs of the present study. Pomegranate buds started hardening earlier than the stem but other reports showed that hardiness begins slightly later in the buds than in the twigs (Sakai and Larcher, 1987). Although both stem and bud of all the genotypes achieved their maximum hardiness level at the same time, pomegranate buds de-hardened earlier. Sakai and Larcher (1987) reported that de-hardening occurs first in the flower buds, then in the leaf buds, and finally in the stems.

Although the hardening process resulted in increased levels of soluble carbohydrates, amino acids, and some fatty acids, responses to low temperature were not the same for the stem and buds of the pomegranate tree. The number of changed metabolites in the stem was twice that of the buds during the hardening period, suggesting that the stem is more influenced by frost stress, compared to buds, thus it is more prone to alteration at metabolic levels. The higher levels of amino acids in the stem compared with buds may be a part of the pre-adaptation strategy, ensuring a higher level of tolerance and guarantee protection against frost stress during winter. Schmitz et al. (2014) suggested that lateral buds were hydraulically isolated from the parent stem during winter until a few days before budburst. We suggest that buds may act as independent parts when compared with branches, reflecting their different behavior in response to frost.

Sucrose, fructose, and glucose were accumulated in both pomegranate stem and buds, suggesting that the accumulation of soluble carbohydrates is a common response of different organs of the tree. The majority of amino acid groups exhibited several times the accumulation in the stem when compared with buds, reflecting that the stem employs more amino acid pathways to improve stress tolerance. Concerning fatty acids, the changes (increase or decrease) in the stem were greater than the buds during the hardening stage. Among fatty acids, stearic acid has a different accumulation profile: its content increases in the stem, and decreases in buds of the hardy group, but it has reverse changes in the sensitive group. Although linoleic and linolenic acids represent similar changes in stem (decrease) and buds (increase), the level of their changes is different between hardy and sensitive groups. According to a comprehensive review by He and Ding (2020), unsaturated fatty acids act as ingredients and modulators of cellular membranes in glycerolipids, a reserve of carbon and energy in triacylglycerol, and stock extracellular barrier constituents (e.g., cutin and suberin), acting as precursors of various bioactive molecules (e.g., jasmonates and nitroalkenes), and regulators of stress signaling.

### Pomegranate Tree Responses to Frost Stress Are Genotype-Dependent

Despite the same hardening/de-hardening timing in all the genotypes and both organs, we found genotypic differences in the hardening/de-hardening rate and maximum frost hardiness level; similar to a previous report on 5-year-old pomegranates (Ghasemi Soloklui et al., 2012) and the other woody perennials such as oak (Morin et al., 2007) and beech trees (Lenz et al., 2016). According to this comprehensive discussion by Lenz et al. (2016), this large variability in freezing resistance in mid-winter can result from genetic differentiations, phenological differences, and phenotypic plasticity.

In agreement with the field observations, KD and MH were the hardiest and the least hardy genotypes, with different morphology during next spring and early summer. Despite a previous report by Rajaei and Yazdanpanah (2015) with four sturdy brown scales in dormant buds of pomegranate cv. Rabbab-e-Neyriz, our recent study demonstrated variability in scale number and compression with leaf primordia. The number of scaly leaves in pomegranate buds was also different in hardy and sensitive genotypes. The greater number of scales and their compression, as well as the higher number of protective leaves, can provide the necessary protection for the most sensitive parts of the trees, i.e., the shoot apex and primordial leaves, against freezing stress. This structural evidence supports greater frost resistance in the KD genotype and is concomitant with our metabolic findings. The number of bud scales in *Abies sachalinensis* increased with winter cold. In boreal conifers of Pinaceae, shoot and flower primordia are enclosed by 40 or more scales and/or modified primordial leaves, which increase the winter survival in northern continental climates (Sakai and Larcher, 1987). During the extra-organ freezing mechanism specific to buds, primordia remain stably unfrozen whereas bud scales freeze first, working as an ice sink to withdraw water from the primordia to the scales (Sakai and Larcher, 1987; Endoh et al., 2014).

During this study, some histological differences were also observed between the stem of hardy and sensitive genotypes. KD showed a thicker periderm, more granular, and massive phenolics in outer cortical layers and smaller xylem vessels, as compared with MH stem (data not shown). These structural attributes are indicative of a difference in freezing tolerance between the genotypes and are under more consideration in our lab, in combination with transmission electron microscope observations.

The same metabolite changes did not occur in all the 15 pomegranate genotypes and may be related to variation in the levels of stress tolerance. Our comparison between the hardiest and the least hardy genotypes (KD vs. MH) revealed that although the accumulation of some metabolites was common in both genotypes; the extent of their increases was higher in KD than in MH. These different responses would enable KD to maintain or improve tolerance during frost stress.

Importantly, KD changes more metabolites than MH during the hardening stage, demonstrating the establishment of strong biochemical strategies to cope with frost stress. Among the investigated metabolites, the group of amino acids shows more accumulation, in both fold change and amount, in KD when compared with MH. Concerning the protective roles of amino acids, the higher accumulation of amino acids in frost-tolerant genotypes represents a relation between stress tolerance and the level of amino acids. This result implies that in pomegranate, amino acids and their derivatives have benefits for frost adaptation, in contrast to soluble carbohydrates and fatty acids. Despite the functional similarity of soluble carbohydrates and amino acids in stress tolerance, the reason for this preference is unclear and may be due to the energetic and/or nutritional cost to produce a large amount of metabolites belonging to these groups (Benina et al., 2013).

## Conclusion

In conclusion, pomegranate frost sensitivity was different seasonally, genotypically, and within the tree. The metabolite quantification represented different metabolic adaptations among genotypes and between the stem and bud, indicating genotype- and organ-specific responses which lead to different levels of frost tolerance. We found that the hardening process has a dramatic impact on the content of pomegranate metabolites and that changes in metabolite levels could be important for the development of frost tolerance. The levels of the metabolites increased during the hardening period, but this was not equal between genotypes or plant organs. KD as the most frost-hardy genotype and stem as the most frost-tolerant organ has a high capacity in stress regulation by the greater accumulation of metabolites, particularly amino acids.

Increased knowledge about frost hardiness and underlying mechanisms, the timing and the rate of hardening/de-hardening, the degree and the maintenance of freezing tolerance, as well as its relations to dormancy-growth cycle and seasonal temperature shifts under global warming could impact horticultural production systems. Parallel structural, ultrastructural, transcriptomic, and metabolomic studies on contrasting pomegranate genotypes, currently running in our lab in ABRII, will help gain a more comprehensive understanding of pomegranate freezing response and underlying mechanisms.

## Data Availability Statement

The original contributions presented in the study are included in the article/Supplementary Material, further inquiries can be directed to the corresponding authors.

## Author Contributions

PY and MZ designed the experiment. PY, MZ, and SA performed the experiment. PY, SA, and MG analyzed the data. MZ and PJ supervised the experiment. PY wrote the manuscript. HR revised the manuscript. MV identified the genotypes. All authors contributed to the article and approved the submitted version.

## Conflict of Interest

The authors declare that the research was conducted in the absence of any commercial or financial relationships that could be construed as a potential conflict of interest.

## Publisher's Note

All claims expressed in this article are solely those of the authors and do not necessarily represent those of their affiliated organizations, or those of the publisher, the editors and the reviewers. Any product that may be evaluated in this article, or claim that may be made by its manufacturer, is not guaranteed or endorsed by the publisher.
