# Supplementary material for: Seasonal Metabolic Investigation in Pomegranate (Punica granatum L.) Highlights the Role of Amino Acids in Genotype- and Organ-Specific Adaptive Responses to Freezing Stress
Source: Front Plant Sci. 2021 Aug 12;12:699139. doi: 10.3389/fpls.2021.699139 (PMC8397415; doi:10.3389/fpls.2021.699139)
Supplement: Supplementary file 1 [file Data_Sheet_1.zip › Supplementary_Material/Supplementary_Material.docx]

**Supplementary Material**

**Supplementary Figure 1.** Seasonal changes in minimum air temperature at the experimental site during 2015-2017.

**Supplementary Figure 2.** Principal component analysis (PCA) of metabolites in stem (**A** and **C**) and bud (**B** and **D**) of fifteen pomegranate genotypes. Biplots (**A** and **B**) of samples for the first two components (PC1 and PC2) where the descriptors of importance are given as arrows and where the length of an arrow is a measure of the descriptor’s variance and the angle between arrows is a measure of the correlation between descriptors, with a small angle expressing a high correlation. Genotype names are abbreviated as codes; see Materials and methods. Loading plots (**C** and **D**) of metabolites for the first two components (PC1 and PC2). Each point represents a single metabolite in **C** and **D**.

**Supplementary Figure 3.** Partial least square discriminant analysis (PLS-DA) of metabolites in the stem of contrasting pomegranate genotypes in response to frost stress. **A**: PLS-DA score plots of metabolites in KD and MH at two time points (October and December). **B**: PLS-DA performance measurements. Accuracy, multiple correlation coefficient R2 and the explained variance in prediction Q2 are shown. The red asterisk indicates the best value of selected measure (Q2).

**Supplementary Figure 4.** Partial least square discriminant analysis (PLS-DA) of metabolites in the buds of contrasting pomegranate genotypes in response to frost stress. **A**: PLS-DA score plots of metabolites in KD and MH at two time points (October and December). **B**: PLS-DA performance measurements. Accuracy, multiple correlation coefficient R2 and the explained variance in prediction Q2 are shown. The red asterisk indicates the best value of selected measure (Q2).

**Supplementary Figure 5.** Heat maps of the Pearson correlation coefficient matrix for the frost hardiness (LT50), minimum temperature (T_min_) and investigated metabolites in stem (**A**) and buds (**B**) of all pomegranate genotypes during two developmental cycles. The color scale on the right side shows the strength of the correlation using blue (strong negative correlation) and red (strong positive correlation).
