# Supplementary figures and images for: Seasonal Metabolic Investigation in Pomegranate (Punica granatum L.) Highlights the Role of Amino Acids in Genotype- and Organ-Specific Adaptive Responses to Freezing Stress
Source: Front Plant Sci. 2021 Aug 12;12:699139. doi: 10.3389/fpls.2021.699139 (PMC8397415; doi:10.3389/fpls.2021.699139)

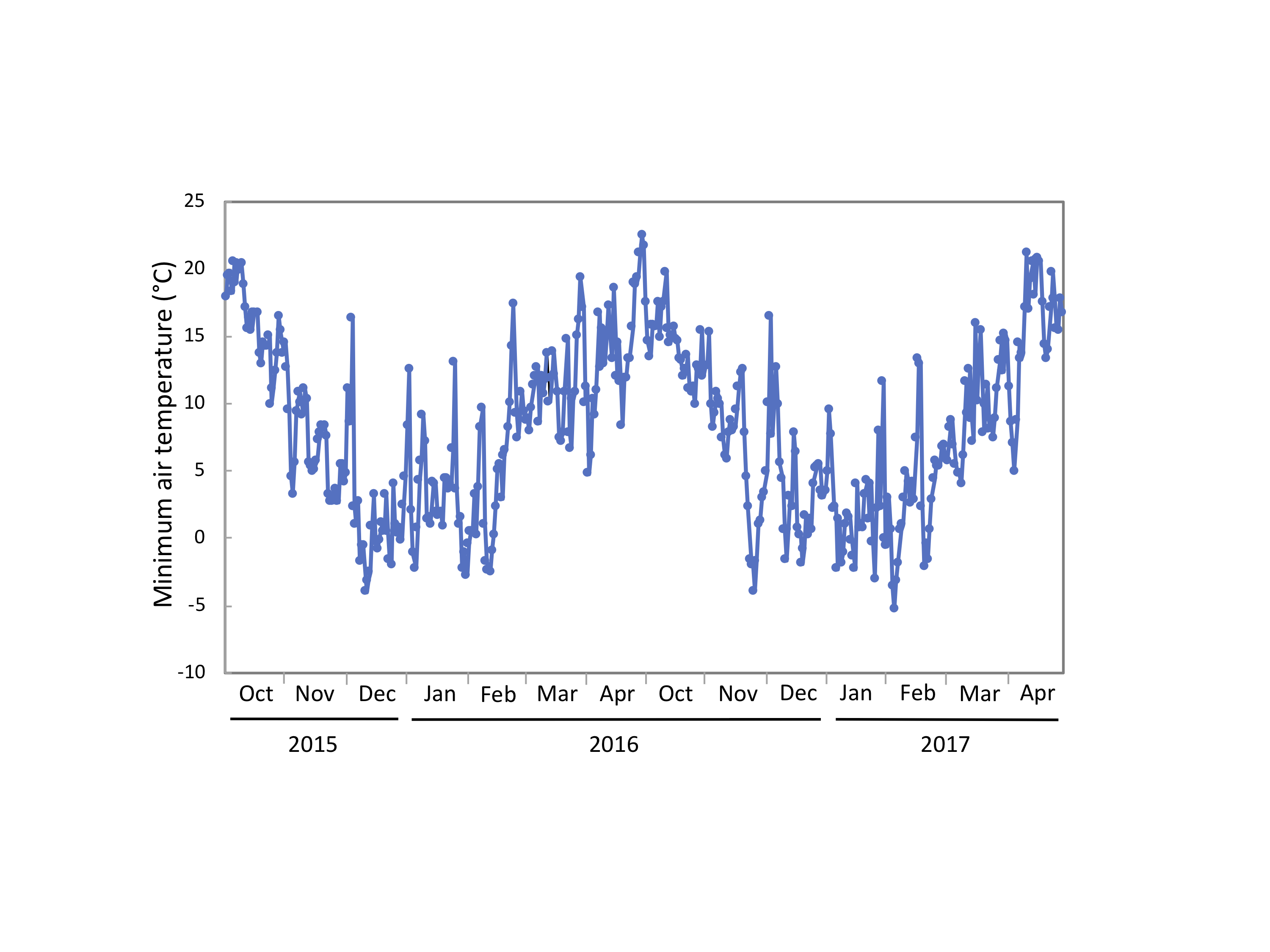

Supplement: Supplementary file 1 [file Data_Sheet_1.zip › Supplementary_Material/Supplementary_Figure_1.tiff]

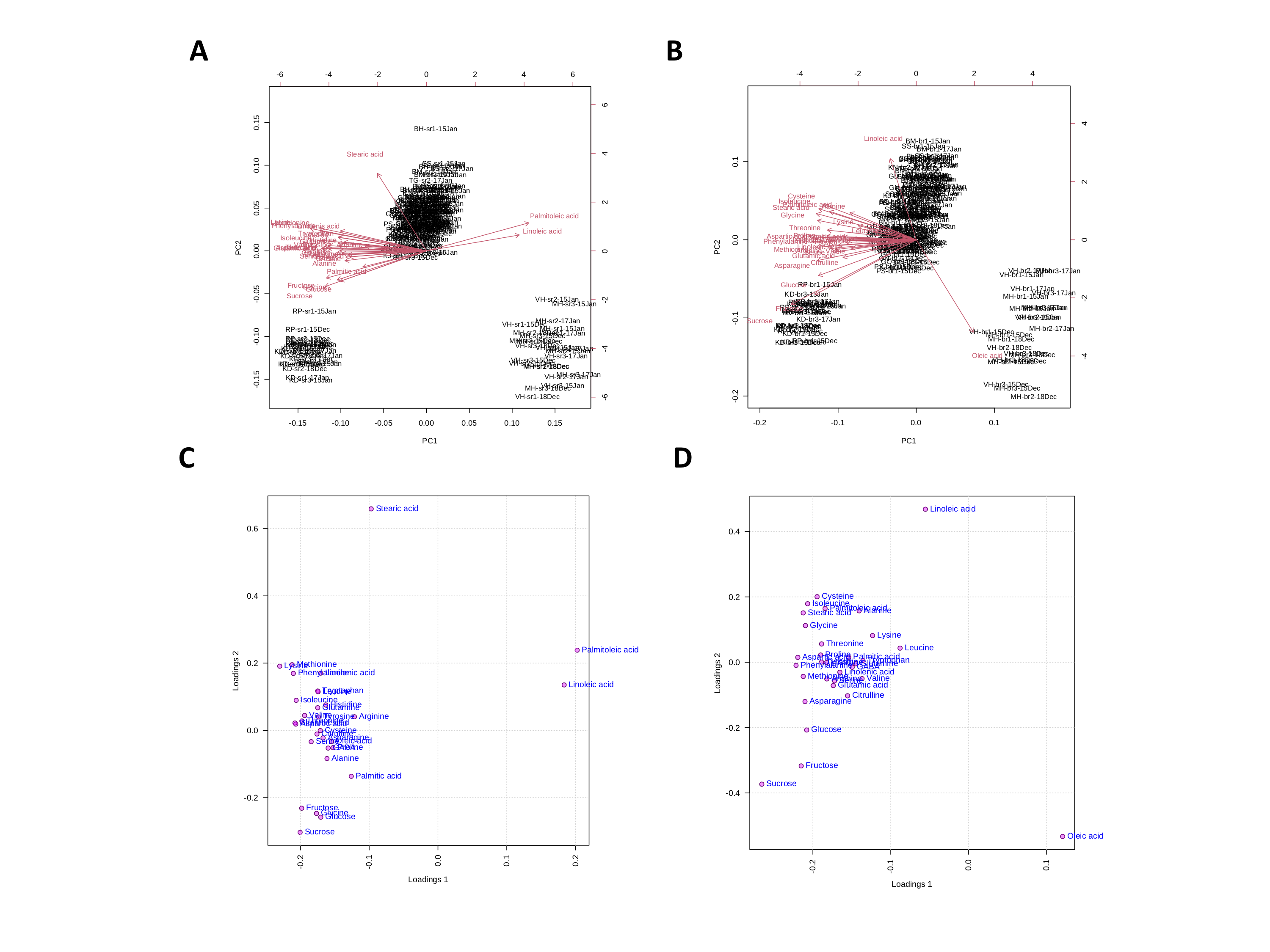

Supplement: Supplementary file 1 [file Data_Sheet_1.zip › Supplementary_Material/Supplementary_Figure_2.tiff]

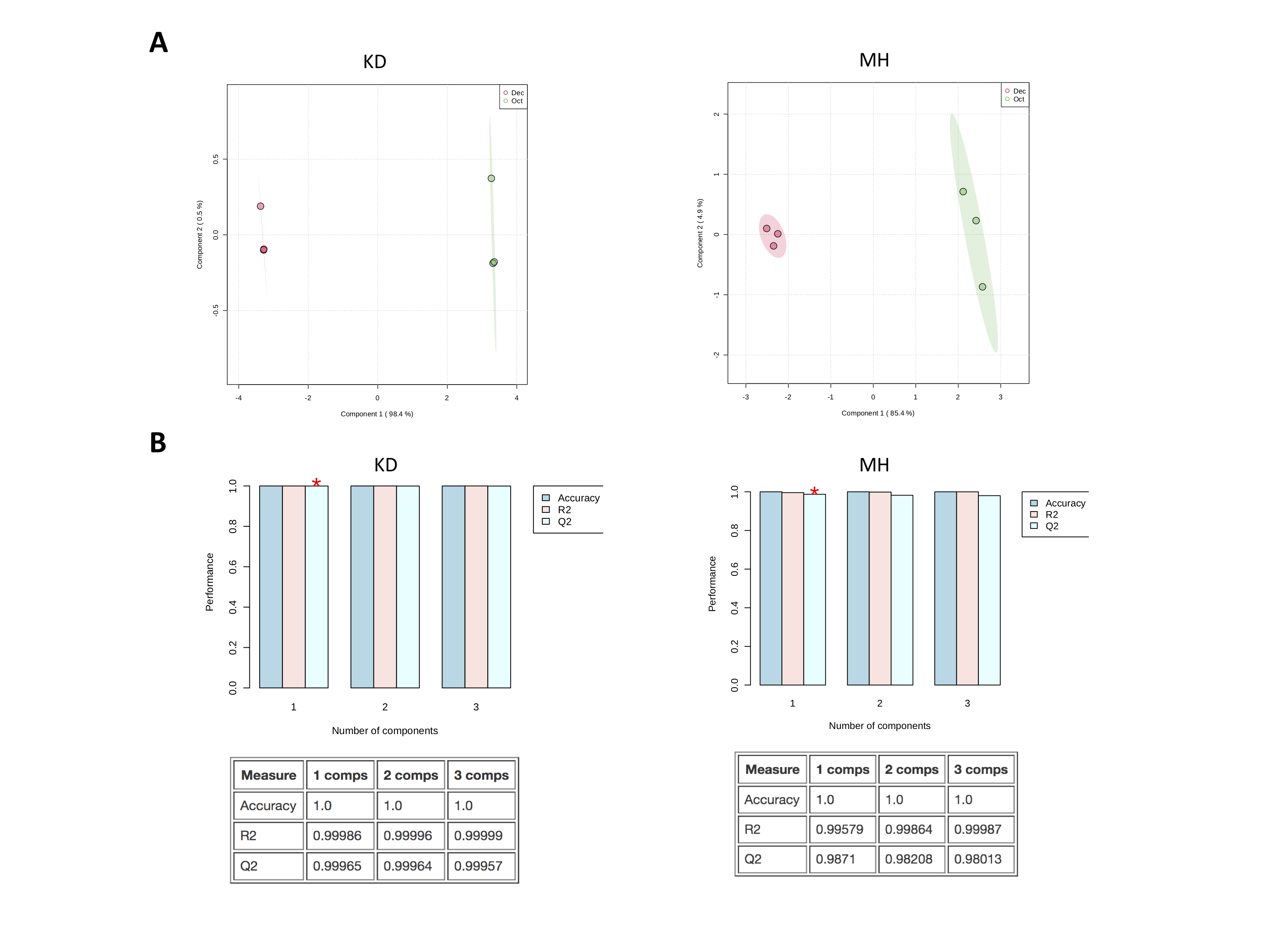

Supplement: Supplementary file 1 [file Data_Sheet_1.zip › Supplementary_Material/Supplementary_Figure_3.tiff]

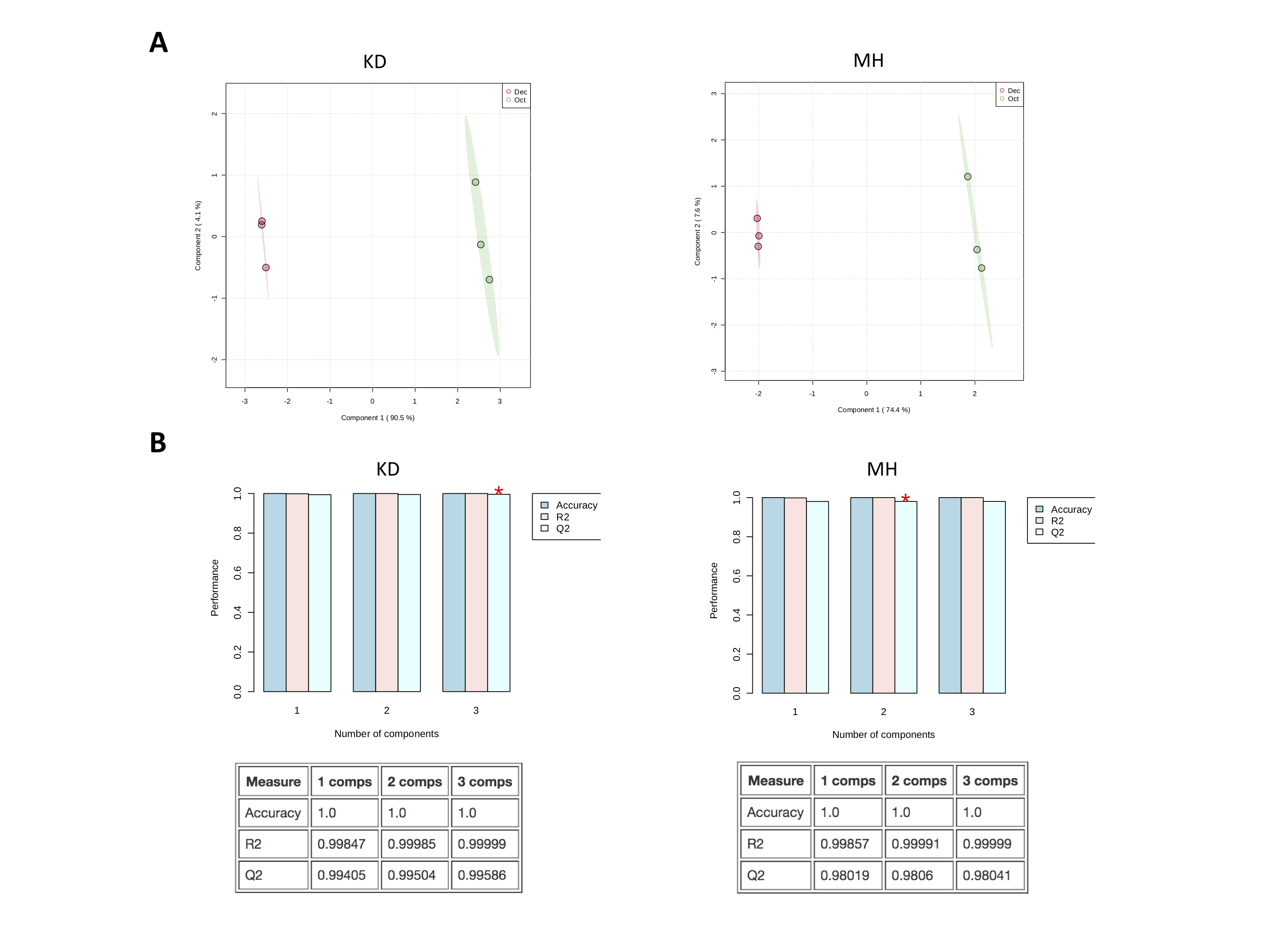

Supplement: Supplementary file 1 [file Data_Sheet_1.zip › Supplementary_Material/Supplementary_Figure_4.tiff]

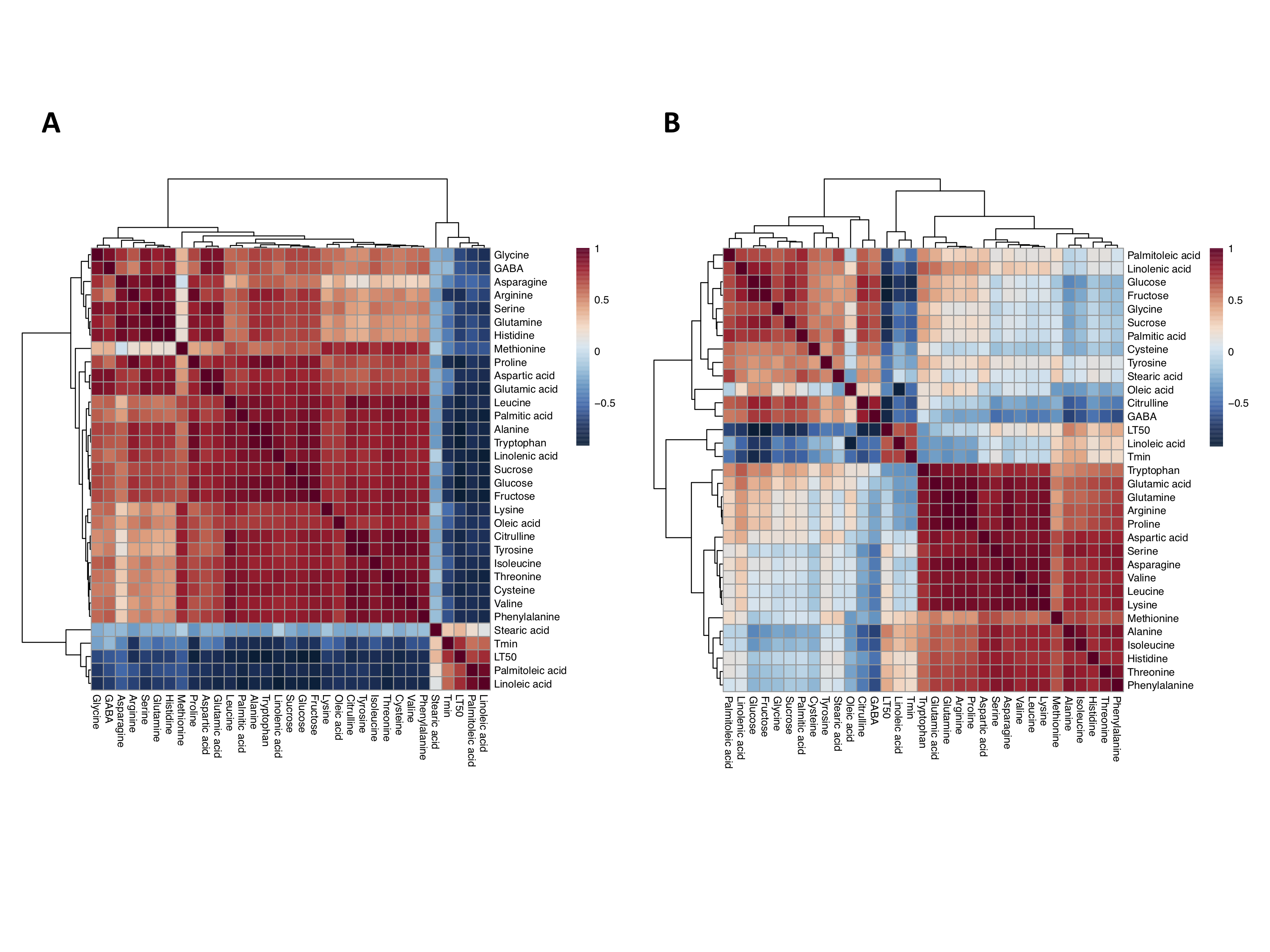

Supplement: Supplementary file 1 [file Data_Sheet_1.zip › Supplementary_Material/Supplementary_Figure_5.tiff]
